# The implementation of safer drug consumption facilities in Scotland: a mixed methods needs assessment and feasibility study for the city of Edinburgh

**DOI:** 10.1186/s12954-024-01144-1

**Published:** 2025-01-13

**Authors:** James Nicholls, Wendy Masterton, Danilo Falzon, Andrew McAuley, Hannah Carver, Kathryn Skivington, Josh Dumbrell, Andy Perkins, Samantha Steele, Kirsten Trayner, Tessa Parkes

**Affiliations:** 1 Institute for Social Marketing and Health, Faculty of Health Sciences and Sport, University of Stirling, Stirling, Scotland; 2 Salvation Army Centre for Addiction Services and Research, University of Stirling, Stirling, Scotland; 3 School of Health and Life Sciences, Glasgow Caledonian University, Glasgow, Scotland; 4 Figure 8 Consultancy, Dundee, Scotland

**Keywords:** Drug consumption, Drug consumption rooms, Drug policy, Harm reduction, Scotland, Needs assessment

## Abstract

**Background:**

Scotland currently has amongst the highest rates of drug-related deaths in Europe, leading to increased advocacy for safer drug consumption facilities (SDCFs) to be piloted in the country. In response to concerns about drug-related harms in Edinburgh, elected officials have considered introducing SDCFs in the city. This paper presents key findings from a feasibility study commissioned by City of Edinburgh Council to support these deliberations.

**Methods:**

Using a multi-method needs assessment approach, we carried out a spatial and temporal analysis of drug-related data in Edinburgh including health, mortality, consumption, crime and service provision indicators; and 48 interviews including 22 people with lived/living experience (PWLE) of drug use in the city, five family members affected by drug-related harms, and 21 professional stakeholders likely to be involved in commissioning or delivering SDCFs. Data were collected using a convergent parallel design. We carried out a descriptive analysis of quantitative date and a thematic analysis of qualitative data. Quantitative data provides an overview of the local context in terms of recorded harms, service provision and consumption patterns as reported in prior surveys. Qualitative PWLE and families data captures the lived experiences of people who use drugs, and affected loved ones, within that local context, including perceived consumption trends, views on the practicality of SDCF provision, and hopes and anxieties regarding potential service provision. Professional stakeholders data provides insights into how people responsible for strategic planning and service delivery view the potential role of SDCF provision within the context described in the quantitative data.

**Results:**

In Edinburgh, drug-related harms and consumption patterns are dispersed across multiple locations, with some areas of higher concentration. Reported levels of opioid use, illicit benzodiazepine use and cocaine injecting are high. Qualitative interviews revealed strong support for the provision of SDCFs, and a preference for services that include peer delivery. However, PWLE also expressed concerns regarding safety and security, and professional stakeholders remained uncertain as to the prioritisation of facilities and possible opportunity costs in the face of restricted budgets.

**Conclusion:**

There is a strong case for the provision of SDCFs in Edinburgh. However, service design needs to reflect spatial distributions of consumption and harm, patterns of consumption by drug type, and expressed preferences for both informality and security among potential service users. Models of SDCF provision used elsewhere in Scotland would therefore need to be adapted to reflect such considerations. These findings may apply more broadly to potential SDCF provision in the UK and internationally, given changing patterns of use and harm.

**Supplementary Information:**

The online version contains supplementary material available at 10.1186/s12954-024-01144-1.

## Introduction

Scotland has amongst the highest drug-related death (DRD) rates in Europe, with 1172 recorded drug deaths in 2023 and rates significantly higher than a decade ago [1–4]. While definitions are not identical, in Scotland there were 224 ‘drug misuse deaths’ per million people in 2023 compared to 22.5 per million people across the European Union in 2022 [1, 2]. In recent years, DRDs in Scotland have been characterised by high levels of polydrug use, including opioids, benzodiazepines, cocaine, gabapentinoids and alcohol [1]. While opioids are implicated in the majority of DRDs in Scotland, in 2023 benzodiazepines were implicated in 58% of deaths and gabapentinoids in 38%. Significant numbers of benzodiazepine-related deaths involve so-called’street benzos’, i.e., benzodiazepines (such as Etizolam and Bromazolam) that are not licensed for prescription and are produced by illicit manufacturers [5]. More recently, concerns have been raised about the increasing prevalence of synthetic opioids in drug markets, especially nitazenes which are significantly stronger than heroin [6, 7].

In response to increasing DRD trends, the Scottish Government has made tackling drug-related deaths a priority by establishing a new treatment strategy in 2018 [8], a Drug Deaths Taskforce in 2019 [9], and a National Mission to reduce drug deaths in 2021 [10]. In July 2023, the Scottish Government published its goals for drug policy in Scotland. Prominent among these is a call for the introduction of Safer Drug Consumption Facilities (SDCFs) [11]. More broadly, a 2023 report by the UK House of Commons Home Affairs Committee called for SDCFs to be piloted in areas where need was identified. Support for SDCFs to be explored in the UK has also been expressed by, among others, the Advisory Council on the Misuse of Drugs [12, 13], the Faculty of Public Health [14], the House of Commons Health and Social Care Committee [15], and the House of Commons Scottish Affairs Committee [16].

SDCFs are variously referred to as supervised injection sites, supervised injection facilities, safer consumption spaces, drug consumption rooms, and overdose prevention centres. They are low-threshold services where people consume pre-obtained drugs in a supervised area with trained staff who can respond in the event of an overdose [17]. In their most basic form, they are usually housed within an enclosed location (either a building, temporary structure or mobile vehicle) which provides hygienic spaces (often booths with a table, mirror and bin) where drugs can be consumed under some form of supervision in case of overdose or adverse reaction. Safe injecting, and in some cases smoking and inhalation, materials are usually supplied and there is usually a space for post-consumption monitoring [18, 19].

More than 200 SDCFs operate globally in at least 12 countries [20, 21] and a growing body of international review evidence demonstrates their effectiveness in addressing a range of key harms [18–23]. While commonly linked to the prevention of overdose deaths, SDCFs have the potential to serve a number of additional purposes. Importantly, they can help to reduce the transmission of blood-borne viruses (BBV) such as HIV and hepatitis B and C, through creating hygienic consumption environments, preventing needle-sharing, and providing effective on-the-spot care for wounds and injuries [24–26]. They can also support people who are significantly marginalised, including from health services, to access wider support both through the provision of on-site harm reduction information and signposting to other services [17, 18, 27, 28]. A core principle of SDCF provision is to provide support that is not judgemental and, thereby, reduces stigma within the service. By providing a compassionate, non-stigmatising environment, SDCFs can also act as a critical point of contact for people who may not otherwise engage with health or wider services [24, 29, 30]. Staff may be able to advise service users of wider support opportunities, materials (e.g. leaflets) can be available, or outreach teams may operate from the service itself. Finally, SDCFs have the potential to reduce public drug use and associated street litter by providing a single, sheltered location where paraphernalia can be securely disposed [31–33].

Until recently, legal barriers meant that there were significant challenges to opening such a facility in Scotland. This changed in September 2023 when the Lord Advocate of Scotland stated, in regard to proposals for an SDCF in Glasgow, ‘it would not be in the public interest to prosecute drug users for simple possession offences committed within a pilot safer drug consumption facility’ [34]. While this statement is explicitly limited to the SDCF proposed for Glasgow, and based on its specific operating and evaluation plans, other cities in Scotland and the rest of the UK are now actively considering opening such facilities [35, 36].

Edinburgh (pop c.550,00) sits in the Lothian health authority region (pop c.916,000) [37]. In 2023, there were 182 recorded drug-related deaths in Lothian, of which 111 were in Edinburgh—approximately a 136% increase since 2010 [1]. In February 2023, following a request from elected members of City of Edinburgh Council, the Edinburgh Alcohol and Drug Partnership commissioned a needs assessment and feasibility study for SDCF provision in the city. The study aimed to address the following research questions:

1. Do patterns and prevalence of drug consumption in Edinburgh contribute to risks that could be mitigated through the provision of SDCF?
2. Do spatial patterns of drug-related harm in Edinburgh indicate optimal locations for the placement of SDCF services?
3. Is SDCF provision supported by people with lived/living experience of drug use (PWLE), affected families and professional stakeholders?
4. What model(s) and / or characteristics of service provision are preferred by PWLE, affected families and professional stakeholders?
5. What challenges or barriers to the implementation of SDCG services in Edinburgh need to be addressed?

Key findings from this study are contained in a report to City of Edinburgh Council, the recommendations of which were approved by the Council in March 2024 [38, 39]. While limited to a single city, our findings provide key insights into the practical challenges of establishing SDCF provision in the context of rapidly developing patterns of consumption. They also provide critical insights into the hopes, expectations and concerns of both people with living experience and affected loved ones, and how these might be addressed in the process of service design. Finally, by addressing the perspectives and concerns of people likely to be involved in commissioning and delivery, the research adds further depth to existing studies that describe the political, strategic and operational considerations that need to be addressed in navigating this terrain [30].

## Materials and methods

### Study design

We carried out a multi-methods study, in which quantitative and qualitative data were collected using a convergent parallel design. Routine and administrative data were collected from a range of sources relating to the population of interest (PWLE) in Edinburgh, including current service provision and a range of drug-related harm indicators (Table 1; see Supplementary Material for complete details of each data set). These descriptive statistics were aggregated at as granular a level as possible without compromising anonymity. Each source was extracted to Excel and, where possible, ranked by order to identify areas with highest frequency of the given indicator over the time period provided. Simple geospatial analysis was also carried out to identify correlations between geographical units ranking highly on indicators. Geographical units varied depending on data source, including: individual address (safe injecting equipment supply); datazone (DRDs and NFOD callouts); beat area (police incidents); postcode (treatment referrals and drug checking services); and electoral ward (drug litter callout requests) (see Supplementary Material for full details). As a result, only some indicators are directly comparable; however, manual comparison of areas (e.g. identifying where a datazone existed largely within a ward) allowed for broad observations to be made about key areas in the city.

**Table 1:** Administrative data sources

Indicator	Geographical unit	Source	Date range	Notes
Patterns of injecting drug use	Injecting equipment provision (IEP) provider address	Needle Exchange Surveillance Initiative (NESI)	2017–18; 2019–20	Previously published [42] and directly supplied
Drug-related deaths	Datazone (defined areas of 500–1000 population used for small area statistics in Scotland)	NHS Lothian Public Health Intelligence Team	2019–21	Directly supplied
Ambulance callouts for non-fatal overdoses where naloxone was administered	Datazone	Scottish Ambulance Service	2018–21	Pre-published data supplied for a separate study [ACODOS: Ambulance Call-Outs to Drug Overdoses in Scotland: Patterns & Practice. University of Stirling]
Postal drug checking service results	Postcode district	Welsh Emerging Drugs and Identification of Novel Substances (WEDINOS) [38]	2014–22	Directly supplied
Injecting equipment provision	Provider address	Lothian Harm Reduction Team NEO 360 database	2020–22	Directly supplied
Drug treatment referrals	Postcode district	NHS Lothian Analytical Services	2019–22	Directly supplied
Hep C tests in drug and alcohol services	Testing location	NHS Lothian Analytical Services	2019–22	Directly supplied
Public requests for drug-related litter removal	Ward	City of Edinburgh Council Environmental Team	2019–22	Directly supplied
Incidents logged as drug-related in police records	Beat area and ward	Police Scotland	2019–22	Directly supplied
Willingness to use an SDCF	IEP provider address	Needle Exchange Surveillance Initiative (NESI)	2017–18	Previously published [42]

Qualitative interviews were conducted with 49 participants. These consisted of PWLE (n = 22: 15 men and seven women); family members (FM) affected by drug-related harms (n = 5; four women and one man); and professional stakeholders (PS) working in the city (n = 22; 14 men and eight women). JD and SS conducted all interviews with PWLE and family members; AP conducted all interviews with professionals. JD is a professional with lived experience within the Edinburgh community and was known to a number of participants; SS had practical experience as a specialist support worker in the region. The involvement of researchers with lived experience, as well as the professional experience of researchers working closely within the service provision landscape, added considerable strength to both data gathering and analysis. Our topic guides were informed by that experience, and reflected what the team understood to be key concerns among both potential service users and providers. Furthermore, we were able to recruit among some participant groups more effectively than may have been the case for a research team not perceived as knowledgeable within the relevant communities. However, we also recognised that personal familiarity with interview participants brings a risk of response bias, and we sought to mitigate this through careful development of topic guides and consistency in interview approaches. We also engaged reflexively, through scheduled meetings throughout the data gathering and analysis process, on how associated professional and personal relationships had the potential to influence our interpretation and framing of responses.

Participants were recruited using a combination of purposive, snowball, and convenience sampling methods to ensure a diverse and representative sample from the target populations. PE participants were purposively recruited from existing professional networks and services including statutory (e.g. NHS) and third sector (e.g. charity providers) health, social care, housing, and criminal justice services. Senior staff known to the research team were contacted and invited to interview. They were also invited to propose alternative or additional participants for us to contact. Staff include people responsible for both operational oversight, such as day-to-day planning and strategic decision-making, such as commissioning. PWLE participants were recruited from two Edinburgh-based third sector organisations (a homelessness crisis support service, and a substance use harm reduction and outreach service) who, following initial contact and meetings, agreed to support the study. FM participants were recruited through a family group open to all individuals from the Midlothian area affected by a loved one’s substance use in Edinburgh. Recruitment strategies included placing informational posters in services catering to the target populations, as well as direct invitation by research staff. Planning meetings were held with staff within the supporting services, and they agreed to informally refer eligible and interested clients. Interested participants made themselves known to service staff or the recruitment team in person or by phone. PWLE and FM participants were eligible for inclusion if they were recent attendees at the recruitment site services; there were no further exclusion criteria. There was no fixed limit set for the number of participants; rather we sought to recruit as large a number as possible within the time period available.

Data were collected through semi-structured interviews, using three topic guides (one for each of the participant groups) developed by the research team. The guide for PWLE participants included questions on current drug use; perceptions of drug use in the city; perceptions of what SDCF provision involved; views on the potential value of SDCF service provision; and views on practical considerations (e.g. accessibility, staffing etc.). The guide for FM participants was broadly the same, but without questions on current use. The guide for PE participants included questions on perceptions of what SDCF provision involved; views on potential value of provision; and views on strategic opportunities and challenges regarding implementation. All PWLE interviews were face-to-face and carried out at a homelessness crisis centre in Edinburgh city centre. PWLE interviews reported a range of drug using experiences, with most injecting either currently or previously and a wide range of substances including heroin, crack, powder cocaine, benzodiazepines and alcohol being consumed. FM and PE interviews were conducted via phone, Zoom or Microsoft Teams. Interviews were recorded and lasted between 20 and 60 min. PWLE and FM participants were offered £20 as either a bank transfer or cash. Interview data was collected between July and October 2023.

Recordings were transcribed for analysis by a professional transcription service. Consent was provided in writing by all participants. Field notes capturing contextual observations and reflections were taken by JD and SS and reviewed during debriefing sessions. Transcribed data were analysed using a framework analysis approach, with codes and themes developed both deductively (based on the topic guides) and inductively (arising independently from the data) [40]. Deductive codes included themes contained in topic guides such as location and perceived benefits; inductive codes included, for example, security and prioritisation. Following familiarisation and initial code development, charting and indexing was carried out via a coding matrix in Excel. PWLE and FM data was coded by JD and JN, with input from SS and AP. PE data was coded by AP and JD, with input from JN. Emerging themes within and between the coded interview datasets were discussed by the research team, and key findings and interpretation agreed prior to write-up.

All quantitative data were non-identifiable, and figures were not reported where numbers were less than five in any given geography to avoid identification of individuals. Ethical and governance approvals for qualitative data collection were obtained from the University of Stirling NHS, Invasive or Clinical Research Panel (NICR, ref. 14024), with additional approvals from NHS Lothian Research and Development Department (ref. 2023/0053) for interviews with NHS staff. Ethical approval was not required for collation of quantitative data as these were gathered and anonymised by the providers and shared with approval from the relevant data teams.

## Results

In presenting our results below, descriptive analysis of routine data is provided under ‘indicators of harm’. The section headed ‘patterns of consumption’ also contains quantitative data, but as this theme emerged in our analysis of qualitative material we include material from interview data here as well. Remaining qualitative findings are set out under the topic headings, which reflect key emergent themes in our interview data: ‘perceived value of SDCF provision’; ‘staffing and security’; ‘location and operating hours’; and ‘integration and prioritisation’. Where it provides additional contextual information, quantitative data is included alongside qualitative material.

### Indicators of harm

**Fig. 1:**
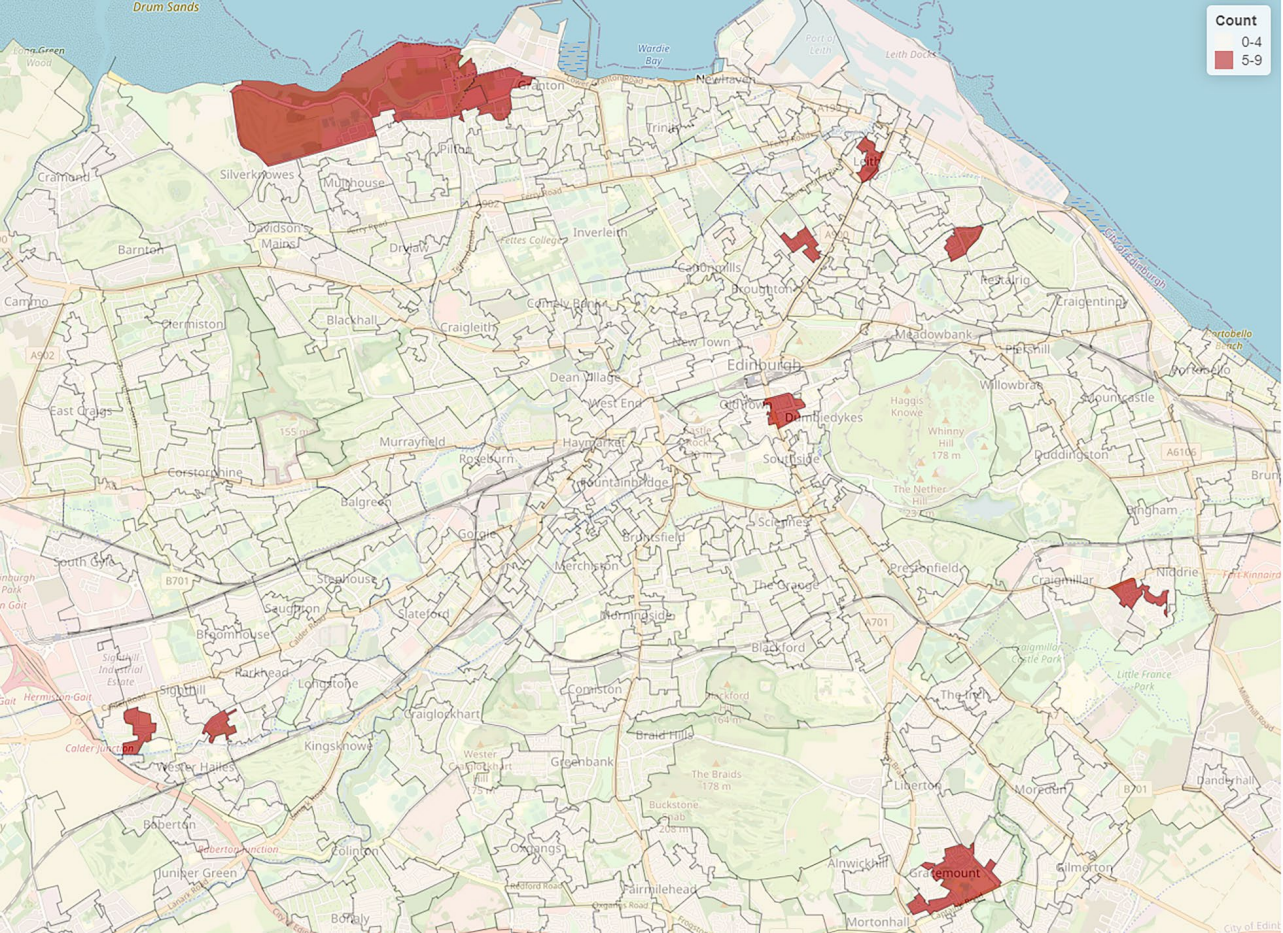
Total drug-related deaths by datazone 2019–21 (Source: NHS Lothian Public Health Intelligence Team)

**Fig. 2:**
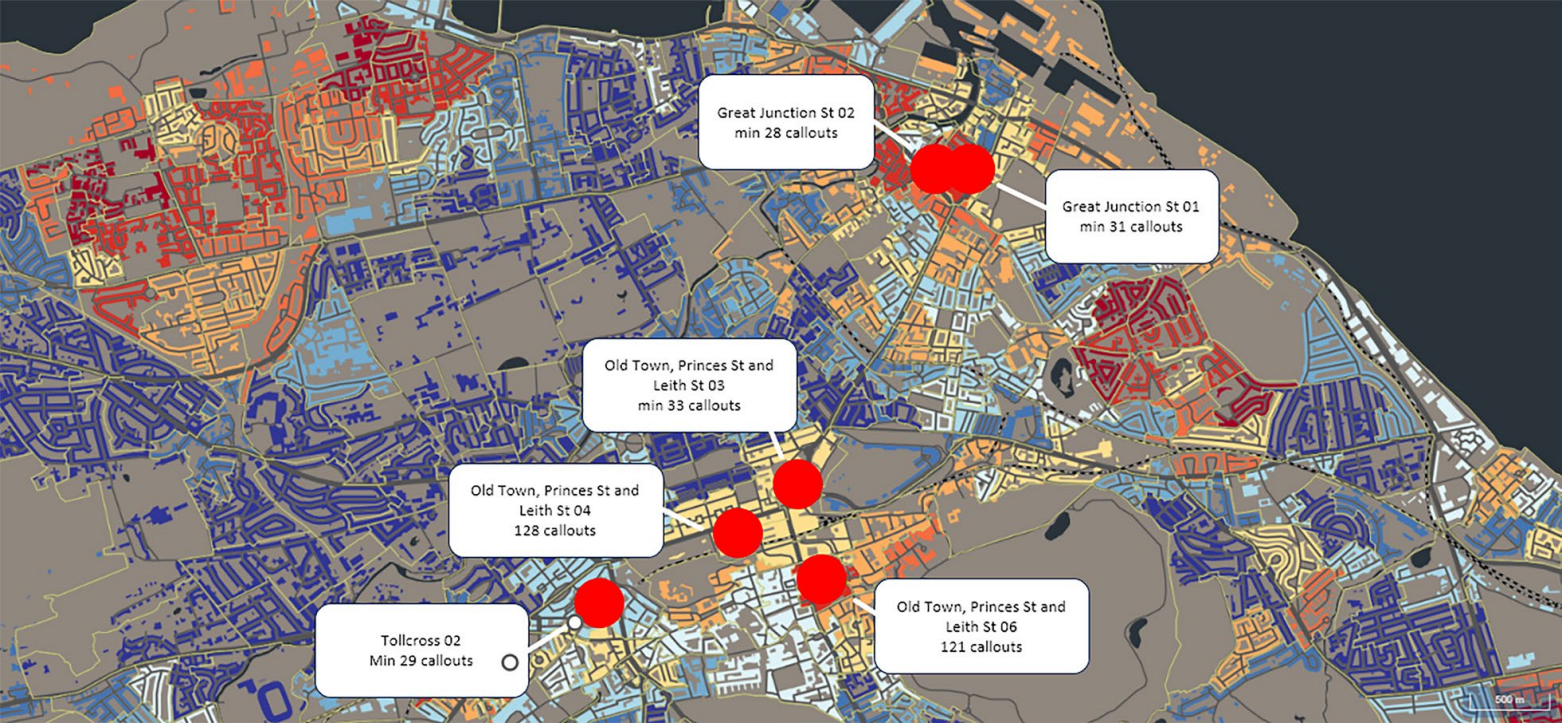
Edinburgh datazones with minimum of 25 + NFOD callouts 2017–21 (Source: Scottish Ambulance Service)

Data for DRDs is collated by NHS Lothian Public Health Team, who provided the research team with the map in Fig. 1. Ambulance callouts for non-fatal overdoses (NFODs) in the city were provided by the Scottish Ambulance Service as part of a separate Scotland-wide research study [41], and are used with permission here. Figure 2 was created using open-source mapping software. NB: the numbers of non-fatal overdoses are considerably higher than fatal, and the same individual may be the subject of a NFOD callout multiple times. DRDs and ambulance callouts for NFODs were aggregated at datazone level. Datazones are the smallest geography used for spatial statistical analyses in Scotland, with populations of between 500–1,000 people. The available data show that both DRDs and NFODs were dispersed across the city, with no single area of uniquely concentrated harms. Figure 1 shows datazones that experienced more than five DRDs between 2019 and 21, revealing a dispersed distribution and clusters in both the city centre and certain outlying districts. When aggregated to larger postcode districts, those with the highest numbers of DRDs included areas of the city that are ranked in the most deprived quintiles according to the Scottish Index of Multiple Deprivation.

Figure 2 shows the datazones with at least 25 NFODs between 2017 and 22 (NB: where datazones saw less than five callouts in a single year, this could be any number between one and five). This shows clusters of harm in the city centre and a small number of nearby areas (e.g., Leith).

Police Scotland’s drug-related incidents data include a wide range of events, including, for example, ‘strong smell of cannabis’, ‘man injecting in stairwell’, ‘drug dealing’, ‘bag of drugs found’ etc. They therefore reflect a wide variety of incidents recorded by police, many of which do not lead to follow-up action, and many of which (e.g. those associated with cannabis) will be of little direct relevance to SDCF provision. Nevertheless, these figures show a geographical correlation between areas of highest recorded incidents and areas with high numbers of both DRDs and NFOD callouts. Importantly, however, police incident figures are not objective indicators of behaviour as they also reflect police priorities and strategic planning (Table 2).

**Table 2:** Police beat areas in Edinburgh with highest number of drug-related incidents 2021–2022

Beat area	Ward	Locality	Number of incidents		
			2021	2022	Total
NW24	Leith Walk	North East	118	229	347
CE21	City Centre	South East	146	134	280
SN37	Southside/Newington	South East	141	67	208
CE20	City Centre	South East	122	66	188
PC48	Fountainbridge/Craiglockhart	South West	85	101	186
PW51	Sighthill/Gorgie	South West	88	94	182
NF07	Forth	North West	125	51	176
NL27	Leith	North East	92	77	169
CE22	City Centre	South East	68	90	158
NW29	Leith Walk	North East	82	66	148

City of Edinburgh Council provides a service for public reporting of drug-related litter. Data from 2019 to 22 also showed the highest levels of reported litter in the city centre and parts of Leith. An important caveat to this is that these higher numbers may reflect that residents in those areas are more likely to file reports meaning the measure is not objective. However, the areas with the highest number of discarded drug litter reports correlate closely with areas where more objective measures of drug-related harm (DRDs and NFODs) are also concentrated (Table 3).

**Table 3:** Edinburgh ward areas with more than 25 requests to CEC for removal of discarded needles 2019–22

Ward name	Locality	2019	2020	2021	2022	Total
City Centre	South East	53	22	19	41	135
Leith Walk	North East	15	26	19	23	83
Leith	North East	8	16	16	14	54
Southside/Newington	South East	12	14	8	6	39
Sighthill/Gorgie	South West	9	8	8	12	37
Craigentinny/Duddingston	North East	7	5	9	6	27
Forth	North West	3	7	8	8	26
Pentland Hills	South West	6	4	7	9	26

### Patterns of consumption

Survey data collected for Public Health Scotland showed high levels of opioid, benzodiazepine, and cocaine use (including injecting) among drug service users in the city [42]. In 2017–18, nearly a quarter of survey respondents who inject drugs accessing drug treatment services in Edinburgh reported powder cocaine or crack cocaine injection. Data on drug-checking results for the city were directly provided to the research team by the WEDINOS (Welsh Emerging Drug and Identification of Novel Substances) service, which tests samples posted from across the United Kingdom. This showed considerable levels of adulteration: of 213 submissions to WEDINOS from Edinburgh in Jan-Oct 2022, 33% contained substances other than expected by the purchaser (private communication).

Interview participants also highlighted the diversity of drug consumption in the city. Many reported widespread benzodiazepine use and cocaine injecting, in addition to crack smoking and heroin use:

*Everybody is going for cocaine. One minute there was no cocaine in Edinburgh, now it’s flooded. A lot of folk have started to inject it, or just wash it back [to make crack] and smoke it now. So, yeah [...] cocaine has gone through the roof in Edinburgh*. [Interviewee 11, Living Experience, Male]

*I tell you, that's probably been the biggest like outbreak I've seen in the last couple of years with people injecting prop* [high-strength cocaine]*. It's scary how quick it's built up […] It's an epidemic, aye, it's definitely that. It's actually shook the town to be honest. [Interviewee 15, Living Experience, Male]*

Widespread polydrug use has been a known issue in Scotland for a number of years, and is implicated in the majority of drug-related deaths [1]. These patterns of use were also reported by our participants.

*[Illicit] Valium: it's such a problem in the city. And then you've got a lot of cocaine users and you've got a lot of people using intravenously with cocaine now as well and mixing a whole cocktail of drugs. People are just using a multitude of stuff*. [Interviewee 09 - Operational, Third Sector, Criminal Justice]

Our research revealed a dynamic and evolving drug scene in Edinburgh. 'Street benzos’ and cocaine injecting were seen by participants as both exacerbating the ongoing drug death crisis and creating a range of novel challenges.

Many participants commented on the challenge of providing a facility that could address the diverse needs and behaviours of people taking a range of different drugs. There was a strong sense that what works for opioid injecting may not be the same as for cocaine injecting or benzodiazepines. For instance, ‘chill out’ spaces might be used very differently or not used at all:

*You’ve got folks who take the Valium, right. They might go into a room and they’re just gouched out. They just want to sleep there. Are you going to have room… is there going to be rooms? […] I don’t know, because with the crack, to be honest with you, with crack, it’s quite easy. You can smoke and it’s not a downer. So, for me, if I was going to use the room, I would be in and out.* [Interviewee 04, Living Experience, Male]

It was recognised that this had implications for service design and the range of harm reduction interventions on offer. The reality of multiple drug use was viewed by some participants as also making the case for on-site drug-checking:

*The* [drug checking] *facility for me is really important […] because obviously we've got a high number of people using benzos as well. So, at least if they could get their benzos checked, that would be amazing.* [Interviewee 12—Operational, Public Sector, Health]

Overall, participants described a complex scene in which a wide variety of drugs were being consumed, often together. The precise contents and purity of substances was not always clear, increasing uncertainty around the effects of use on any given occasion. Consumption patterns were dynamic, and this had implications for the kind of services and support being provided in facilities.

### Perceived value of SDCF provision

SDCF provision was supported across our participant groups. However, different benefits were emphasised, as were the relative values of what provision might achieve. The potential for SDCFs to address acute overdose risks was acknowledged by all participants and seen as fundamental to their value:

*I've lost so many mates and like everybody in my life died with overdoses. It's crazy. But a drug consumption room: I just see it as somewhere clean and safe for people to go and inject, take drugs. It's more about the safe part because so many overdose and I've noticed nobody has a fuckin’ clue what to do when it happens*. [Interviewee 15, Living Experience, Male]

SDCFs were also viewed as playing a vital role in improving the safety around injecting practices. This was partly about the provision of sterile equipment, but also about providing the time and space for injection to be carried out safely. Injection outdoors was viewed as especially risky since it often involved both unhygienic conditions and the need to complete the process quickly:

*Anywhere else I've gone, I'm having to rush things. I'm not cooking up properly. I'm missing where I shouldn’t be missing because I'm rushing, so that's where the abscesses come from*. [Interviewee 07, Living Experience, Male]

Consuming drugs outdoors was also associated with shame and stigma. Participants described experiencing both physical risks associated with homelessness (such as being attacked or robbed) and psychological risks arising from daily experience of trauma, stigma, and extreme economic insecurity. Many saw SDCFs as potentially mitigating these wider challenges through the creation of non-stigmatising environments where a sense of community could be fostered:

*It’s somewhere that you don’t have to sit in the street cold or watching over your back because you’re scared of getting seen by certain people, or hiding in car parks or parks. It’s just a place you can go and you won’t be judged [...] Yeah it is a safe place. A safe haven for people that are drug users. Because I don’t think they’ve ever had that, really.* [Interviewee 19, Living Experience, Female]

The perceived value of SDCFs, in this respect, was not only to prevent overdose and promote safer consumption practices, but also to allow a space in which the pressures of persistent stigmatisation were reduced, and positive social environments could be developed.

### Staffing and security

Participants held a range of views on what models of staffing would best address the needs of potential service users. Informality and accessibility were seen as critically important, with many sharing the view that the ideal model would be a ‘*really low barrier, high tolerance service. Like, not clinical. Not NHS-type service.*’ [Interviewee 14—Operational, Third Sector, Homelessness]. Achieving this required integration of peers in the design and delivery of services:

*Because* [peers] *are the only people that understand, truly understand. I don’t care how many books you've read and how many seminars you've sat through and how fuckin’ many times God's spoke to you and told you to go on this path. I don’t care about all that. It makes absolutely no blind bit of difference.* [Interviewee 07, Living Experience, Male]

Peer delivery was viewed as reducing moral judgement, and supporting empathy with the day-to-day experiences of service users. By extension, this approach would support the creation of a non-stigmatising environment. A number also felt that peer workers could also act as role models:

*Like somebody who is maybe a recovered addict who can… because that’s always helped me […] I've always liked hearing success stories. That's always given me a bit of hope that it can be done.* [Interviewee 01, Living Experience, Male]

Peer delivery, and the integration of peers into service design, was widely viewed as an essential component of effective provision. There was a strong sense that the experience, knowledge and compassion brought by peers would not only increase the attractiveness of a service, but ensure provision was well-informed and better able to respond to potential challenges.

However, clinical expertise and formal governance were also viewed as important by many participants. It was felt by many that a key characteristic of SDCF provision was support and supervision by people who had the formal clinical skills to deal with medical emergencies, and that this factor was important to both establishing trust and mitigating risk:

*So, controlled environment is controlled environment. That means […] certain educated people, especially chosen for that. They're going to supervise them; they're going to look after them.* [Interviewee 03, Living Experience, Male]

There was also a clear view that peer delivery should not be viewed as simply a cheaper alternative to formally trained staff. Participants were conscious that peer roles would be challenging and potentially stressful. There was a need for proper recognition, in terms of both remuneration and wellbeing support, of the work involved. This was seen as vital to supporting a triangulation of skills across lived and learnt knowledge:

*We could have volunteers as well, but people who bring that experience. Gone are the days where we can expect them to do that for nothing. This isn’t charity. This is really important work, so we have to invest in them.* [Interviewee 10—Strategic, Public Sector, Health]

The challenge of managing risks was raised by several participants. Many PWLE participants expressed concerns around how unpredictable behaviours could be managed in such a way as to support the safety or comfort of both service users and staff. In particular, there were concerns around behaviours associated with cocaine and benzodiazepines, which could create specific problems:

*They're quite irresponsible when they're on these benzos, so I don’t know if it will work in that kind of controlled environment because how are you going to control them?* [Interviewee 03, Living Experience, Male]

*Well, a lot of people get paranoid when they take that coke as well. They'd just leave straightaway*. [Interviewee 14, Living Experience, Male]

Participants also identified several risks to clients not directly linked to consumption itself, including the risk of violence, theft, or the fear that ‘*people might try and use this opportunity to sell drugs’* [Interviewee 19, Living Experience, Female]. Although consumption in an SDCF was widely expected to be safer than elsewhere (especially compared to public spaces), many participants expressed concerns that, without clear rules and safeguarding procedures, an SDCF could still be a risky environment. A strong theme arising from interviews was the need for measures to protect staff and clients, including clear regulations and protocols:

*Because it’s going to be a drug consumption room, someone is going to come here to use their drugs. So, you might have people that don’t have drugs watching for: “Oh, he must have drugs—I’m going to rob him”. He might… you know what I mean? That type of thinking.* [Interviewee 04, Living Experience, Male]

While risk management and safeguarding were important concerns, so too was the issue of formal police involvement. There was a lack of trust in the police and a fear that service use could increase the risk of arrest. Many stated that limiting police involvement, and ensuring confidentiality within the service, would be essential for fostering trust:

*Well, nobody will get involved if the police are going to get involved [...] I am not going to go there if I’m going to get stopped by the police.* [Interviewee 02, Living Experience, Male]

Overall, the need to be responsive to the range of consumption and associated risks was seen as having implications for staff training, service design (e.g. provision of inhalation rooms), and risk assessment. There was broad support for a model of delivery that recognised the central importance of lived experience in creating non-stigmatising, trauma-informed environments and the need for staff who intimately understood the experience of drug use; but which also benefitted from the knowledge and skills brought by staff with professional training. The two were seen not as in conflict, but as mutually beneficial.

### Location and operating hours

While participants saw value in SDCF provision, the reality of travelling to a set location was viewed as a significant problem. If the service was close to where they lived, or where they usually consumed drugs, then it was of much more benefit than if it was at a distance. Noticeably, very few PWLE participants discussed proximity to place of purchase, but this is a further plausible consideration. In principle, however, travelling a long way to use a service was simply not seen as realistic by a number of participants.

*Should there not be a few of them located through Edinburgh because there can't just be the one? … are people going to be getting on the bus and just going up to* [the SDCF]*… Aye, that's what I mean: “Fuck it, I'm not going on the bus. I'm going away for a hit”. If they're using prop* [cocaine] *it's: "I'm not fuckin’ going way up there" type of thing, “fuck that.” [...] So, people from the town I could see coming here, but not people from the schemes [council housing estates].* [Interviewee 15, Living Experience, Male]

While some participants concluded that a city centre location would be the most pragmatic option, others argued for multiple sites across the city although they recognised this would not entirely resolve the problem of travel, which would remain a barrier for people living at a distance from areas served by a facility. Some felt no location would fully address the problem of travel times, especially for people who used more frequently:

*Would I have sought out a safe consumption room? Would I have scored my drugs in the city and then went maybe down to Leith or something, wherever it is, and travel to a safe consumption room to use safely? I don’t think so.* [Interviewee 09 - Operational, Third Sector, Criminal Justice]

No participant viewed the question of location as easily resolved. As an alternative, some suggested a ‘high tolerance’ model in which, rather than creating a discrete SDCF, existing services, such as homeless hostels, allowed consumption on their premises while having provisions in place to respond to emergencies:

[In] *my experience of working in homelessness environments, third sector staff are doing overdose prevention every single day […] I think there's something about acknowledging the greater tolerance that a lot of homelessness services have now. For example, the service that I'm in now has a vending machine for injecting equipment. We have naloxone that we're giving out. There's discussions about how to keep safe. We do wellbeing checks.* [Interviewee 14—Operational, Third Sector, Homelessness]

Overall, there was agreement that any solution to the problem of location would involve a degree of compromise. Edinburgh was not viewed as a city with an obvious ‘open drug scene’ which provided a natural location for a facility, and the routine data gathered for the study supported this perception. The majority of respondents did not, however, see this as a reason not to establish provision. Instead, they viewed it as strengthening the case for more flexible models. Notably, PE participants in particular felt that the costs and logistics of mobile provision meant it would be impractical. However, a number highlighted the potential value of an implementation design that combined formal consumption spaces with high tolerance approaches in services such as sheltered housing.

### Integration and prioritisation

In addition to direct benefits, participants emphasised the importance of safer consumption facilities providing services that extend beyond acute support. A number noted that SDCFs should not simply involve coming ‘*in one door and out another*’ [Interviewee 21—Operational, Public Sector, Social Work]. Rather, they should be integrated with wider harm reduction support and signposting to services:

*It has to be more than just a place where people can come to access clean equipment and to be able to use safely. It has to be that gentle step into other services.* [Interviewee 14—Operational, Third Sector, Homelessness]

A number of FM participants also emphasised the value of SDCF provision as supporting pathways to wider services, including treatment.

*The other thing is trying to get people involved with the services to obviously reduce, and then eventually go into some kind of treatment format. I think [SDCFs] have to be linked with other services as well so that people aren't seeing it as like a public house where you just go along and you order, or whatever.* [Interviewee 24, Family Member, Female]

The perceived relationship between SDCF provision and wider services was complex, and no single perspective dominated. Some participants emphasised the importance of harm reduction on its own merits, while others emphasised integration and signposting as a key component. This was further complicated by the issue of opportunity costs and limited budgets across the drug sector. Despite the high levels of support in principle, participants (especially those working in funding and commissioning) were mindful of spending implications in the context of extremely challenging finances. In this context, several felt that while SDCF provision could make a valuable contribution other interventions remained a higher priority.

*I wouldn’t put the* [SDCF] *above a very well-funded treatment system. And if you told me I had to choose between the two, I would have a very well-funded treatment system.* [Interviewee 16—Strategic, Public Sector, Commissioning]

At the same time, some PWLE participants made the case that the severity of the crisis necessitated provision and that SDCFs could reduce treatment costs further down the line:

*There's a lot of people are dying out there. If something like that was there, they wouldn’t be dying.* [Interviewee 06, Living Experience, Male]

*Number one because everything else comes after that.* [Further support] *wouldn’t be needed as much if this was an option to start with*. [Interviewee 08, Living Experience, Female]

No participants viewed SDCFs as a panacea for drug harms, or as an isolated solution. As one participant stated: *‘it's not just about safe injecting facilities or safe consumption facilities or overdose prevention sites. You need to have good welfare, good quality housing provision.’* [Interviewee 06—Strategic, Third Sector, Homelessness]. Although seen by many participants as valuable, and by some as critical, SDCFs were not viewed by any as a ‘silver bullet’. Rather, they were seen as an important element of a wider response to a drug harms crisis, and an intervention that should be conceptualised as part of an integrated system that draws on prevention, harm reduction, treatment, and recovery.

## Limitations

Local data, both directly supplied and published, was aggregated at different geographical levels. This makes direct comparisons difficult and means that some of the geographies considered (e.g., postcode areas) cover locations with significantly different socioeconomic and harm profiles. However, both DRDs and NFOD ambulance callouts data were available at datazone level, and these represent the core objective measure of direct harms used in our analysis. As discussed above, police incident data reflect operational practice, which we were unable to analyse independently, and drug litter data are prone to reporting bias as they are dependent on calls to the council hotline. We treated both datasets cautiously as a result, only using them to provide contextual information. People who experienced homelessness and who use, and inject, drugs represent one of the primary targets of SDCF provision. However, because our sample of PWLE only included people accessing a homelessness service, their views may not be transferable to other communities in Edinburgh or more widely.

## Discussion

Edinburgh city faces a range of challenges related to drug consumption and harm, including areas with high concentrations of drug-related deaths, ambulance callouts for non-fatal overdose, discarded needles and drug-related police incidents. This data also shows areas where combinations of harms are concentrated. International review evidence for SDCFs [17–23] suggests that they can play a key role in: reducing the risk of overdose for those consuming in the facility; supporting safer injecting practices among people attending facilities; providing harm reduction advice for people attending facilities; signposting and / or referring attendees to wider social support and treatment services; reducing drug litter in the vicinity and improving public amenity; and tackling stigma and promoting compassionate care. Among our participants, there was extensive support for SDCF provision on the basis that it would achieve some or all of these outcomes. This reflects earlier Scottish studies showing broad acceptance among decision-makers that international evidence is sufficient to justify trial implementation [30], a perception among affected family members that SDCFs represent a key opportunity for positive change [29], and a high willingness to use such services among people who inject drugs [43].Importantly, support was also based on the belief that the provision of compassionate, non-judgemental services had intrinsic harm reduction benefits—something that has been identified as key in a number of studies [17, 20, 43–46].

International studies demonstrate that SDCF service models vary considerably [17, 20, 21, 38, 47, 48], and approaches to staffing also range from predominantly voluntary to highly specialised and professional [44, 45, 47, 49, 50]. There is extensive evidence of service user support for peer delivery, [17, 35, 45, 46, 49, 51] but also, in line with our findings, that access to clinically trained professionals is valued and may increase engagement with health, social, and drug-treatment related support [17, 28, 49]. We found that it was less a question of peer delivery versus clinical staffing, and more a nuanced discussion around the appropriate balance of roles and functions within a mixed staffing model. Such considerations not only concerned the experience of service users, but the provision of adequate training and support for staff, given the potential for stress and burnout that is both identified in the research literature and highlighted by a number of our participants [17, 20, 44, 52, 53].

Both survey and interview data point to high levels of cocaine and benzodiazepine use, including cocaine injection, alongside heroin use. Increasing risks from drugs other than opioids are not unique to Edinburgh. Cocaine injecting was a key factor in an outbreak of HIV infections among people who inject drugs in Glasgow in 2015 [54], and cocaine was the most injected drug in an unsanctioned mobile overdose prevention centre that operated in Glasgow from 2019 to 21 [55]. The emergence of synthetic opioids such as nitazenes in the United Kingdom drug supply is also cause for serious concern [6, 7, 56].Similar issues are likely in other cities where decision-makers are considering implementation, though the precise patterns and types of drug use will vary. SDCF design should not, therefore, be based on assumptions that the majority of use will be opioids, or that provision should be built solely around consumption needs, and physiological or behavioural responses, associated with opioids. Rather, design decisions need to be made in response to local needs and, ideally, with sufficient flexibility to address changing trends in terms of substances consumed and modes of administration.

While routine data on harms showed clear areas of high concentration, they do not suggest a single standout location for an SDCF. Furthermore, interview participants expressed the view that a single central location was at best a pragmatic compromise. This suggests that multiple, smaller sites may be more appropriate than a large, standalone facility in this city. While a number of high profile SDCFs are standalone sites, integration within existing services (e.g., as a dedicated space within a local treatment or harm reduction service) is the most common model of provision globally, and one which may reduce costs by drawing on shared resources [26, 57–64]. That said, we also found support for considering in-service provision as part of a ‘high tolerance’ model in, potentially, multiple sheltered accommodation locations. While mobile sites exist in a number of cities [47, 57, 64, 65], they were not seen as practical by participants in this study. However, mixed delivery that included both standalone services and high tolerance approaches in housing services was viewed by some as a positive approach.

Participants were realistic about the challenges involved, including balancing informality and peer-delivery with clinical expertise, careful governance, and safeguarding for both staff and service users, especially where multiple drug types were involved. There was also a recognition that, without additional funding, and in the context of a city facing an array of developing challenges, SDCF provision involved significant opportunity costs. While all participants saw at least some value in SDCF provision, not all supported prioritising SDCF provision over other interventions or activities. In the context of severely limited funding, the question of whether to introduce SDCFs was not one of principle but relative priorities [30, 66].

There is extensive global evidence on the role SDCFs can play in signposting to wider services and their capacity to provide a point of contact for those who may not otherwise engage with those services [17, 24, 27], though signposting alone does not guarantee engagement especially if services are over-subscribed [51]. Participants viewed SDCF provision as one element in the wider harm reduction and treatment landscape. While views were mixed on this (e.g. some felt they could reduce the need for treatment, others felt they could provide a first step), there was no sense that SDCFs should be seen as isolated from, or as an alternative to, treatment interventions. Rather, their value was as a contribution to a broader, holistic response to an ongoing crisis of drug-related harms, albeit one that had gained political salience because of the legal and political barriers that existed.

Our study was designed to support evidence-informed decision-making on this topic in the city of Edinburgh. In doing so, it also highlights the importance of considering local conditions regarding patterns of use and harm. Not all settings, even within the UK, face the same patterns of consumption and harm identified in the routine data, or types of lived experience described by interview participants, and what is likely to work in one place may not be appropriate in others. However, our findings draw particular attention to some key considerations that are likely to apply elsewhere. For instance, the need to not assume that opioids will be the default, or even primary, drug type used in a UK facility at this point in time. Service models based on addressing the needs of people using opioids may struggle if the majority of attendees (or the large majority of consumption events) involve stimulants and / or benzodiazepines. Our study also highlights the diversity of views among people with living experience on this topic. Not all had the same perspectives or opinions regarding staffing, design, security or integration with wider services. Some were more sceptical than others about the range of benefits a service might bring, or whether they would use the service themselves. This emphasises the need to acknowledge viewpoint diversity among PWUD, and to recognise that no service model will meet the needs and preferences of all potential service users. Similarly, the views of potential decision-makers varied, especially in regard to prioritisation. It was clear that support for SDCF provision in principle was not the same as believing it should be prioritised over other interventions. In this sense, decision-making was less about interpretation of evidence, and more about weighing up opportunity costs. Gaining support for SDCF provision may be less about demonstrating that services ‘work’, and more about demonstrating how they add value to a wider harm reduction and treatment landscape such that the diversion of limited funds is justified.

The challenges in establishing SDCFs in UK cities are considerable, due to legal constraints, changing patterns of consumption, competing funding priorities, and varying levels of political support. Nevertheless, in the context of a continuing drug-death crisis, and the increasing penetration of novel substances into drug markets, there remains a strong case for provision. This study found widespread support among both potential service users and key decision-makers. We would therefore suggest that debate on this topic moves from high-level arguments on global evidence for effectiveness towards details of how services might be best configured to meet local needs and respond to dynamic consumption patterns.

## Supplementary Information

Additional file 1 (DOCX 28 kb)

## Data Availability

Information on the sources for, and location of, data collected for this paper is set out in Table 1 and Supplementary Material. Authors may provide further information on request, subject to approval from the data providers.
