# Correction: The implementation of safer drug consumption facilities in Scotland: a mixed methods needs assessment and feasibility study for the city of Edinburgh

**DOI:** 10.1186/s12954-025-01187-y

**Published:** 2025-03-15

**Authors:** James Nicholls, Wendy Masterton, Danilo Falzon, Andrew McAuley, Hannah Carver, Kathryn Skivington, Josh Dumbrell, Andy Perkins, Samantha Steele, Kirsten Trayner, Tessa Parkes

**Affiliations:** 1 Institute for Social Marketing and Health, Faculty of Health Sciences and Sport, University of Stirling, Stirling, Scotland; 2 Salvation Army Centre for Addiction Services and Research, University of Stirling, Stirling, Scotland; 3 School of Health and Life Sciences, Glasgow Caledonian University, Glasgow, Scotland; 4 Figure 8 Consultancy, Dundee, Scotland; 5 School of Health and Wellbeing, University of Glasgow, Glasgow, Scotland

**Correction: Harm Reduction Journal (2025) 22:6** 10.1186/s12954-024-01144-1

In this article [1], the affiliation for the author Kathryn Skivington was incorrectly given as ‘School of Health and Life Sciences, Glasgow Caledonian University, Glasgow, Scotland’ but should have been ‘School of Health and Wellbeing, University of Glasgow, Glasgow, Scotland’.

The original article has been corrected.
