# Supplementary material for: The implementation of safer drug consumption facilities in Scotland: a mixed methods needs assessment and feasibility study for the city of Edinburgh
Source: Harm Reduct J. 2025 Jan 13;22:6. doi: 10.1186/s12954-024-01144-1 (PMC11730151; doi:10.1186/s12954-024-01144-1)
Supplement: Supplementary file 1 — Additional file 1 (DOCX 28 kb) [file 12954_2024_1144_MOESM1_ESM.docx]

**Routine data sources provided for analysis – extended description**

| **Document section** | **Data source** | **Data source description** | **Indicator(s)** | **Time period** | **Geographical information** | **Limitations of data source** |
| --- | --- | --- | --- | --- | --- | --- |
| Epidemiology of injecting drug use | The Needle Exchange Surveillance Initiative (NESI) | Cross-sectional bio-behavioural survey of PWID conducted across mainland Scotland every two years. PWID are recruited from harm reductions services and provide a dried blood spot test to measure BBV infection and complete a questionnaire to measure demographics, injecting risk behaviours, and other injecting and social risk factors | The epidemiology of injecting drug use and drug-related harms in Edinburgh | 2017-18, and 2019-20 | Data gathered at 15 recruitment sites in the City of Edinburgh. | All data (apart from BBV status) is self-reported, which may be subject to response and recall bias. Recruitment covers only those attending services that provide injecting equipment, and thus may not fully represent the PWID population in Scotland. No data on HIV diagnoses due to low numbers and protection of anonymity. Data collection for 2019-20 was suspended early due to COVID-19, which impacted sample size |
| Drug-related deaths | NHS Lothian Drug-related Death Annual Report (2021); and National Records of Scotland data (NRS) | The NHS Lothian Drug-related Death Annual Report provides figures for Edinburgh and the Lothians aggregated to postcode districts. NB there is a difference in the definition for drug-related deaths between NHS Lothian and NRS. NRS counts deaths only where drugs were listed as the first primary cause of death, whereas NHS Lothian includes all primary drug-related deaths. | Drug-related deaths in Lothian (and Edinburgh specifically); demographics; and drugs implicated. | 2019-21 | DRDs by Edinburgh postcode district. Also aggregated into four Health and Social Care Partnership (HSCP) sub-group localities (Edinburgh North East; Edinburgh North West; Edinburgh South East; and Edinburgh South West) | Due to the small number of deaths when broken down into postcode level, some data were suppressed to protect anonymity and the postcode was not reported |
| Scottish Ambulance Service (SAS) non-fatal overdose callouts | ACODOS study | Ambulance callouts where naloxone is administered to reverse an opioid overdose (Scottish Government, 2021). Incidents recorded by ambulance staff (paramedics and ambulance technicians). | Non-fatal overdose callout numbers | 2018-21 | Datazones with 5+ NFOD callouts in at least one calendar month in a year were mapped. Also aggregated to HSPC localities | For datazones with fewer than five callouts, the exact number was not recorded and therefore could not be accurately included in the amalgamation across HSPC localities. |
| Drug checking | Welsh Emerging Drugs and Identification of Novel Substances (WEDINOS); Public Health Scotland Rapid Action Drug Alerts and Response (RADAR) quarterly reports | WEDINOS receives postal samples for testing from across the UK. Public Health Scotland RADAR reports provide Edinburgh-specific drug warnings | Contents of substances submitted for drug testing from Edinburgh city | WEDINOS data from Jan 2014-Oct 2022 analysed to track trends. Data from Jan-Oct 2022 was drawn on to provide analysis of specific drug market trends. RADAR reports from 2023 were analysed | Where possible, data were categorised by postcode district | Due to small numbers, many findings could not be categorised by postcode district in order to protect anonymity. |
| Injecting equipment provision (IEP) | NEO 360 data available from Public Health Scotland's IEP report. Edinburgh-specific data collated by Lothian Harm Reduction Team | The Injecting Equipment Provision in Scotland report provides NEO 360 data showing IEP services and uptake across Scotland. NEO 360 is a commercially available database used by NHS Boards to record and monitor IEP activity. | IEP data (number of clients, number of transactions, substance per transaction, client demographics) from 10 most active IEP provision locations | 2020-22 | Data from the IEP in Scotland report provided at a health board level. Edinburgh-specific data categorised by pharmacy/service name and postcode | IEP datasets may have missing values, uncertainties, or inaccuracies. |
| Treatment referrals | Specialist drug treatment referral data from NHS specialist addiction services, cross-checked against OAT data provided by Lothian Analytical Services | Trak data (NHS database) provides specialist drug service referral information, and ILLY data (NHS database) provides data on OAT prescriptions | OAT patients referred to specialist drug services. Specifically:  - patients admitted to hospital with clinical notes reporting injecting drug use   - patients registered at Edinburgh Access Practice (used as a proxy for homelessness)  - patients admitted to hospital with clinical notes reporting injecting drug use *and* registered at Edinburgh Access Practice | 2019-2022 | Data aggregated to postcode district. GP location used as proxy for patient address postcode area. | Patients with injecting drug use in clinical notes may not be currently injecting drugs. Patients registered with Access Practice may not currently be experiencing homelessness. When using raw numbers rather than rate data, postcodes in highly populated areas will inevitably have higher numbers. |
| Blood-borne virus (BBV) testing | Number of Hepatitis C tests (collated by Lothian Analytical Services) | Lothian Analytical Services provided data showing number of Hep C tests in Edinburgh-based services that support people who use drugs | Number of Hep C tests | 2019-22 | Service name and postcode district | Positive Hep C tests not reported due to protect anonymity. Where <5 five tests per year service location not reported. |
| Drug-related litter | City of Edinburgh (CEC) street cleaning needle removal service (collated by CEC Environmental Team) | Requests for the removal of discarded drug litter are received (by phone or email) and recorded by the street cleaning needle removal service | Number of requests to CEC for removal of discarded needles |  | Ward area | Requests do not specify if callout is for one or multiple needles. Numbers reflect calls to council, so measure resident activity not objective level of discarded equipment. |
| Drug-related crime | Drug-related crime data collated by Police Scotland | Data from Police Scotland’s national incident recording system, Storm. A Storm incident is listed as: any matters reported to the Police, which require despatch of a Police Officer to the scene; any matter that the Police will be committed to and will take time to resolve; or any matter reported to the Police which, whilst not requiring a Police Officer to attend the scene, still warrants information being recorded.  Police Scotland possession-specific dataset and supply-specific dataset were also provided and analysed | All drug-related incidents; possession- and supply-related incidents | Storm data: 2021-22; possession/supply data: 2019-22 | Beat areas (geographic areas used by Police Scotland to map districts). Corresponding HSCP localities identified and added manually. | Number of incidents liable to vary due to operational policies, variations in police coverage, use of stop and search etc. |
| Willingness to use a SDCF | The Needle Exchange Surveillance Initiative (NESI) | See above | Willingness to use a SDCF from PWID who were recruited in Edinburgh city NESI sites | 2017-18 | See above | Figures pre-date Covid. Data captures reported willingness, so is hypothetical in regard to actual behaviours. |
